# Design of a Three-Axis Piezoelectric Accelerometer with Wide Frequency, High Sensitivity and Low Cross-Axis Interference

**DOI:** 10.3390/mi17091080

**Published:** 2026-09-14

**Authors:** Po-Chun Chen, Ze-Wen Sun, Sheng-Yuan Chu, Cheng-Che Tsai

**Affiliations:** 1 Department of Electrical Engineering, National Cheng Kung University, Tainan 70101, Taiwan995427522szw@gmail.com (Z.-W.S.); 2 Department of Game and Animation Design, Tung Fang Design University, Kaohsiung 829, Taiwan; tsai480116@gmail.com

**Keywords:** design, accelerometer, low cross-axis, high sensitivity, optimization

## Abstract

A single-structure three-axis MEMS accelerometer often suffers from cross-axis interference, which leads to inaccurate sensitivity and makes it difficult to reliably assess the health condition of the measured object. In addition, achieving high sensitivity often comes at the cost of reduced bandwidth. Because few previous studies have addressed the cross-axis interference of such structures, this study presents the development and optimization of a structural three-axis MEMS piezoelectric accelerometer. A mathematical model was first established, followed by simulation and structural optimization to enhance sensitivity and minimize cross-axis interference while keeping the bandwidth nearly unchanged. This study proposes, for the first time, an improved structure that evolves the L-shaped accelerometer into a composite configuration consisting of a W-shaped cantilever and an additional four-bar proof mass, in order to enhance bandwidth and significantly reduce cross-axis interference. Generally, an increase in sensitivity leads to a reduction in bandwidth; however, by modifying the geometry of the proof mass, the proposed accelerometer achieves a substantial improvement in sensitivity while avoiding a significant loss of bandwidth. The implementation of a triaxial accelerometer can generally be achieved in three ways: using three single-axis accelerometers, realizing three axes through circuitry, or achieving three axes through structural design. Using three single-axis accelerometers results in large volume, heavy weight, and high cost. Circuit-based three-axis implementation cannot detect multi-axis forces simultaneously, whereas structural three-axis design is difficult to realize. This study focuses on designing a triaxial accelerometer based on a structural approach, optimizing it with reference to the most successful examples reported in the literature and progressively addressing the drawbacks of previous structures. First, trapezoidal beams are used instead of rectangular cantilever beams to increase bandwidth. Next, two additional cantilever beams are added to transfer the *Z*-axis sensitivity detection points to reduce cross-axis interference. Finally, the shape of the proof mass is designed to enhance the sensitivities along all three axes. The optimized accelerometer exhibits resonant frequencies of approximately 12.6 kHz, 31.6 kHz, and 11.8 kHz along the *X*-, *Y*-, and *Z*-axes, with corresponding sensitivities of 6.11, 5.44, and 8.85 mV/g, and cross-axis interference reduced to below 15%. The results demonstrate the achievement of a highly accurate and reliable three-axis MEMS piezoelectric accelerometer through structural design and optimization. The proposed design concept may also be beneficial for other piezoelectric-based sensor applications.

## 1. Introduction

An accelerometer is a device used to sense inertial motion by converting the movement of a proof mass under acceleration into a corresponding electrical signal. It can be used to measure physical quantities such as shock, vibration, acceleration, and gravity. Such sensors are widely applied in various fields, for instance, in automobile airbag deployment systems to ensure timely activation of protection mechanisms during collisions, in condition monitoring of machine-tool spindles and motors, and in structural health monitoring of bridges and buildings in civil engineering. Depending on their sensing principles, accelerometers can be classified into three main types: capacitive, piezoresistive, and piezoelectric accelerometers [1].

Piezoresistive accelerometers [2] were among the earliest devices developed for MEMS applications. They mainly consist of a proof mass, cantilever beams, and piezoresistors. The advantages of this type include a simple structure and relatively easy fabrication; however, drawbacks such as susceptibility to external disturbances, high sensitivity to temperature variations, and self-heating of the resistive elements often necessitate temperature compensation circuitry in practical applications. Capacitive accelerometers are known for their high accuracy, stability, and tolerance to temperature, but their limitations include a relatively narrow bandwidth, unsuitability for high-frequency applications, and susceptibility to electromagnetic interference [3,4]. Piezoelectric accelerometers share a similar structural concept with piezoresistive types, with the main difference being that piezoelectric materials replace the piezoresistive elements [5,6]. When subjected to an external force or acceleration, the piezoelectric material generates charges proportional to the applied stress. These charges are collected by electrodes and converted into voltage signals via charge amplifiers or converters, enabling the calculation of acceleration. Piezoelectric accelerometers feature a simple structure, high sensitivity, strong immunity to electromagnetic interference, a high resonance-frequency tolerance, and a broad operational bandwidth; moreover, they can operate without an external power supply, making them one of the most widely used technologies for vibration measurement. MEMS piezoelectric three-axis accelerometers can be applied to the monitoring of unmanned aerial vehicles (UAVs) and other flying vehicles, as they are lightweight and compact, require no external power supply, and are capable of measuring multi-axis motion during flight.

In the current market, piezoelectric accelerometers are predominantly bulk-material based [5], offering high sensitivity and wide bandwidth but suffering from large size and heavy weight, which conflict with the trend of miniaturization. Commercial MEMS piezoelectric accelerometers are still rare and generally exhibit inferior performance. Nevertheless, if structural design can significantly improve the performance metrics of MEMS accelerometers, their miniaturization advantage could make them competitive replacements for bulk-type devices.

Most commercial triaxial accelerometers are composed of three individual uniaxial units, which results in a larger size, heavier weight, and higher manufacturing cost. Some academic studies have demonstrated triaxial measurement through circuit-based designs; however, this approach cannot detect multi-axis forces simultaneously [7]. Both methods, therefore, are limited in achieving true multi-axis detection. To address this issue, the present study focuses on a structural design approach that enables simultaneous multi-axis sensing, aiming to enhance the performance of existing triaxial accelerometers by preserving their advantages while mitigating their drawbacks. Numerous studies have investigated MEMS piezoelectric three-axis accelerometers, most of which adopt a cross-shaped structure [8]. The cross-shaped accelerometer typically realizes triaxial measurement by using an external circuit to connect the sensing elements in series or parallel, thereby compensating for the signals from different axes. In 2016, M.-H. Xu et al. designed an X-shaped accelerometer and compared it with the conventional cross-shaped type, finding that the X-shaped design exhibited higher sensitivity and a lower resonant frequency under the same dimensions [9]. In 2021, Y. Liu proposed the first structurally realized triaxial accelerometer, a novel L-shaped structure, which not only achieved a high resonant frequency of 11.7 kHz along the *Z*-axis but also enabled direct three-axis measurement without any additional external circuit [10]. In 2024, F. Khayat further improved the L-shaped accelerometer by modifying its cantilever beams, enhancing the sensitivities of the *X*- and *Y*-axes by approximately 1.5 times and that of the *Z*-axis by about six times [11]. The main drawback of structurally designed three-axis MEMS accelerometers is the cross-axis interference among the different axis signals, which leads to inaccurate sensitivity and prevents accurate assessment of the health condition of the measured object. Moreover, achieving high sensitivity often comes at the cost of reduced bandwidth. However, few previous studies have focused on addressing this issue.

Based on the literature survey, the L-shaped accelerometer in [10] is chosen as the reference in the present work, owing to its superior performance compared with other published results. Therefore, this study focuses on the design and simulation of three-axis piezoelectric MEMS accelerometers, aiming to achieve miniaturization, high performance, and cost-effectiveness, thereby enhancing their potential in vibration-sensing applications. PZT, one of the most widely used and best-performing piezoelectric materials, is adopted in this work because it possesses superior piezoelectric properties and has been studied extensively for more than 50 years [12]. Specifically, a mathematical model of the structure is first derived, followed by sensitivity enhancement through model-based optimization and, finally, the reduction of excessive cross-axis interference. The outcome is an improved MEMS piezoelectric triaxial accelerometer with optimized performance.

## 2. Design Procedure

### 2.1. Undamped Free-Vibration Eigenfrequency Formulation

**Figure 1:**
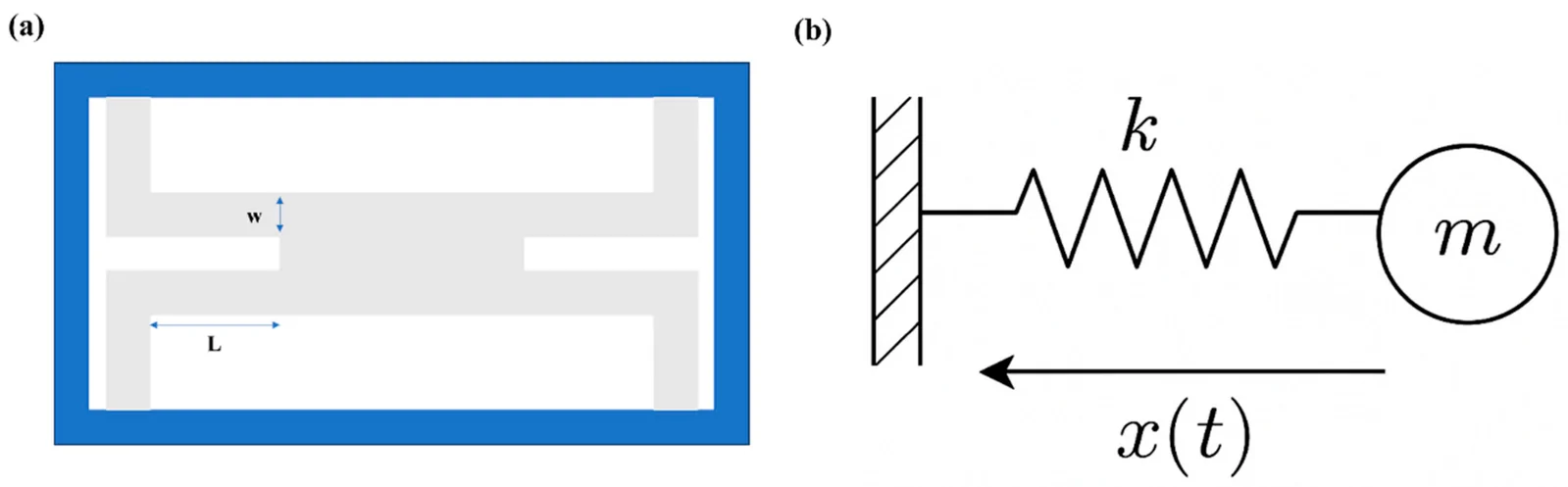
(**a**) Accelerometer structure. The blue region represents the fixed frame, while the gray region represents the device body. (**b**) Simplified spring–mass model.

Before designing the accelerometer, the mathematical model of the resonance frequency is derived. The geometry of the accelerometer to be analyzed is shown in Figure 1a. To simplify the analysis, the structure can be reduced to an equivalent spring–mass model without damping, as illustrated in Figure 1b, for subsequent mathematical formulation. The accelerometer is represented by a reduced-order model using the generalized-coordinate vector

q = [z, θ_x_, θ_y_]^T^,

where z represents the translational displacement along the *Z*-axis, while θ_x_ and θ_y_ represent rotations about the *X*- and *Y*-axes, respectively. The general equation of motion is

$$\left[M]\ddot{q}+[C]\dot{q}+[K]q=F(t\right)$$

**Figure 2:**
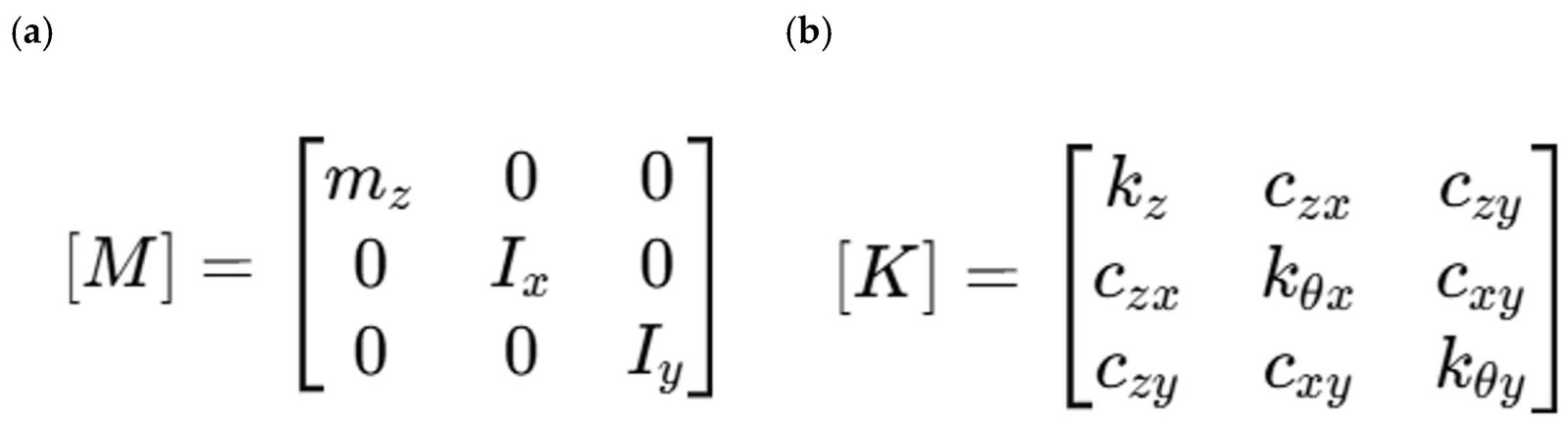
(**a**) Matrix [M]. (**b**) Matrix [K].

In Figure 2a, [M] is the mass matrix describing the inertial properties of the system, where m_z_ represents the effective translational mass along the *Z*-axis, and I_x_ and I_y_ represent the effective mass moments of inertia about the *X*- and *Y*-axes, respectively. In Figure 2b, [K] is the stiffness matrix describing the elastic restoring characteristics of the structure, where k_z_ is the translational stiffness along the *Z*-axis, k_θx_ and k_θy_ are the rotational stiffnesses about the *X*- and *Y*-axes, respectively, and c_zx_, c_zy_, and c_xy_ represent the coupling stiffnesses between different translational and rotational degrees of freedom. Since the coordinate origin is assumed to be located at the center of mass and the rotational axes are aligned with the principal inertia axes, the mass matrix can be simplified as a diagonal matrix. In contrast, because elastic deformation of the suspension structure may cause interactions between different degrees of freedom, off-diagonal coupling terms appear in the stiffness matrix. To determine the undamped natural frequencies, damping and external excitation are neglected, resulting in

$$[M]\ddot{q}+[K]q=0$$

By assuming a harmonic solution q(t) = Φe^jωt^, the governing equation becomes

([K] − ω^2^[M])Φ = 0

Therefore, the natural frequencies are determined from

det([K] − ω^2^[M]) = 0

and the i-th natural frequency is given by

f_i_ = ω_i_/(2π)

### 2.2. Mathematical Model

In this subsection, the resonance-frequency formulas for the *X*-, *Y*-, and *Z*-axes are derived. For simplicity, coupling effects are neglected in the analysis of each axis, so that the motion along one axis does not affect the others.

**Z-axis resonance frequency**

For the *Z*-axis resonant frequency, according to Newton’s second law $F=ma=m\ddot{Z}$ (where acceleration is the second derivative of displacement with respect to time) and Hooke’s law F=−kZ, the accelerometer at resonance is in dynamic equilibrium. Therefore, we can derive

$$m\ddot{Z}=-k_{z}z$$

which gives

$$m\ddot{Z}+k_{z}z=0$$

The corresponding solution and natural frequency are

$$z\left(t\right)=A\cos\left(\omega_{z}t\right)+B\sin\left(\omega_{z}t\right),\;\omega_{z}=\sqrt{\frac{k_{z}}{m_{z}}}$$

$$f_{z}=\frac{\omega_{z}}{2\pi }=\frac{1}{2\pi }\sqrt{\frac{k_{z}}{m_{z}}}$$

Here, k represents the stiffness of the accelerometer, which describes the resistance of the structure to deformation. It is defined as $k=\frac{F}{\delta }$, where F is the applied force and δ is the resulting deformation.

According to the Euler–Bernoulli beam theory, the governing equation is

$$EI\frac{d^{2}y}{dx^{2}}=F(x-L)$$

where E is Young’s modulus of the system, I is the area moment of inertia, and L is the length of the cantilever beam. Integrating twice gives

$$Y=\iint \frac{F(x-L)}{EI}dx^{2}=\frac{F}{EI}(\frac{x^{3}}{6}-\frac{Lx^{2}}{2})+C_{1}x+C_{2}$$

Applying the boundary conditions Y(0)=0 and $Y^{\prime }(0)=0$, we obtain $C_{1}=C_{2}=0$ and thus

$$Y=\frac{F}{EI}(\frac{x^{3}}{6}-\frac{Lx^{2}}{2})$$

Since the proof mass is mainly located at the free end, the displacement at x=L is considered:

(1) $$\delta =Y(L)=\frac{FL^{3}}{3EI}$$

Hence, the stiffness is

$$k=\frac{F}{\delta }=\frac{3EI}{L^{3}}$$

Substituting this into the *Z*-axis resonant frequency equation yields

$$f=\frac{1}{2\pi }\sqrt{\frac{k}{m}}=\frac{1}{2\pi }\sqrt{\frac{3EI}{mL^{3}}}$$

Here, I is the area moment of inertia, which represents the bending resistance of the beam; the larger its value, the higher the bending resistance. It is defined as

$$I=\int y^{2}dA=\int y^{2}w\;dy=w\int_{-h/2}^{h/2}y^{2}dy=\frac{wh^{3}}{12}$$

where w is the beam width and h is the beam thickness.

Finally, substituting I into the frequency equation gives the complete expression:

$$f=\frac{1}{2\pi }\sqrt{\frac{Ewh^{3}}{4mL^{3}}}$$

*X*- and *Y*-axis resonance frequencies

For the *X*- and *Y*-axis resonances, the vibration mode differs from that of the *Z*-axis, as the *X*- and *Y*-axes vibrate primarily in a torsional mode. Therefore, the corresponding equations are also different.

According to Newton’s second law for rotational motion,

$$M=I_{m}\alpha =I_{m}\ddot{\theta }$$

and Hooke’s law for torsional motion,

$$M=-K_{\theta }\theta$$

where M is the torque, $I_{m}$ is the moment of inertia, α is the angular acceleration, $K_{\theta }$ is the torsional stiffness, and θ is the angular displacement.

Under torsional vibration, the accelerometer is in a state of torque equilibrium, leading to

$$I_{m}\ddot{\theta }+K_{\theta }\theta =0$$

By applying the same approach as in the previous subsections, the resonant frequency can be derived as

$$f=\frac{\omega }{2\pi }=\frac{1}{2\pi }\sqrt{\frac{K_{\theta }}{I_{m}}}$$

For torsional vibration, the torsional stiffness is defined as

$$K_{\theta }=\frac{T}{\theta }$$

which represents the torque required to produce a unit angular displacement.

Considering a beam of length L, fixed at one end and subjected to a torque T at the other end, the torsion equation is expressed as

$$\frac{T}{J}=\frac{\tau }{r}=\frac{G\theta }{L_{\theta }}$$

where J is the polar moment of inertia, τ is the shear stress, r is the radius, and G is the shear modulus. The relationship between shear modulus and Young’s modulus is given by

$$G=\frac{E}{2(1+\nu )}$$

where ν is Poisson’s ratio. Rearranging the torsion equation gives

$$K_{\theta }=\frac{T}{\theta }=\frac{GJ}{L_{\theta }}$$

Substituting into the frequency expression yields

$$f=\frac{1}{2\pi }\sqrt{\frac{GJ}{I_{m}L_{\theta }}}$$

Next, we discuss the parameter J. According to Saint-Venant’s torsion theory, for a cantilever beam,

$$J\approx \frac{w_{\theta }{h_{\theta }}^{3}}{3}(1-0.63\frac{h_{\theta }}{w_{\theta }}+0.052{\left(\frac{h_{\theta }}{w_{\theta }}\right)}^{3})$$

When the beam width $w_{\theta }\gg h_{\theta }$, this simplifies to

$$J\approx \frac{w_{\theta }{h_{\theta }}^{3}}{3}$$

which is the case for MEMS piezoelectric accelerometers.

Next, the moment of inertia $I_{m}$ is defined as

$$I_{m}=\iint_{A}r^{2}\rho \;dA=\rho \int_{-a/2}^{a/2}\int_{-b/2}^{b/2}(x^{2}+y^{2})\;dy\;dx=\rho (\frac{a^{3}b}{12}+\frac{ab^{3}}{12})$$

Given that the area A=ab and mass m=ρA, the moment of inertia can be simplified to

$$I_{m}=\frac{m}{12}(a^{2}+b^{2})$$

Finally, substituting J and $I_{m}$ into the resonant frequency equation gives the complete expression:

$$f=\frac{1}{2\pi }\sqrt{\frac{4Gw_{\theta }{h_{\theta }}^{3}}{m(a^{2}+b^{2})L_{\theta }}}$$

For the *X* and *Y* axes, the torque equilibrium relation is used:

$$M=I_{m}\alpha =I_{m}\ddot{\theta }$$

The resonance frequency can then be expressed as

$$f=\frac{\omega }{2\pi }=\frac{1}{2\pi }\sqrt{\frac{k_{\theta }}{I_{m}}}$$

where

$$k_{\theta }=\frac{T}{\theta }=\frac{G\omega_{\theta }h_{\theta }^{3}}{L_{\theta }},I_{m}=\iint_{A}r^{2}\rho dA=\frac{m}{12}\left(a^{2}+b^{2}\right)$$

Finally, substituting the expressions gives the resonance frequency for the *X* and *Y* axes:

$$f=\frac{\omega }{2\pi }=\frac{1}{2\pi }\sqrt{\frac{k_{\theta }}{I_{m}}}$$

### 2.3. ANSYS Data

Simulations were then carried out using ANSYS 2024 R2 software. The accelerometer geometry was drawn in AutoCAD 2023, and the material parameters were applied to both the mathematical model and the simulation. In this study, PZT was used as the piezoelectric layer of the accelerometer [13], while silicon was used for the cantilever beams and the outer frame. The relevant material parameters, including density, Young’s modulus, Poisson’s ratio, d_33_ and dielectric constant, are listed in Table 1.

## 3. Results and Discussion

### 3.1. Model Accuracy

Based on the mathematical model derived in Section 2.2, the calculated resonance frequencies for the *X*-, *Y*-, and *Z*-axes are 41 kHz, 23 kHz, and 12.7 kHz, respectively. To verify the accuracy of the proposed model, the calculated results were compared with both the simulation and the experimental data reported in the literature, as summarized in Table 2. It can be observed that for the *Z*-axis, the simulation and experimental resonance frequencies are relatively close, while the theoretical result obtained from the mathematical model also shows a small deviation. For the *X*- and *Y*-axes, experimental results were not available in the literature, but the comparison with the simulation results shows a level of discrepancy similar to that observed for the *Z*-axis, confirming the validity of the developed model. The observed deviations are likely attributed to factors such as mesh size in the simulation and the approximations made during the theoretical model derivation.

### 3.2. Structural Design and Optimization

**Before optimization**

**Figure 3:**
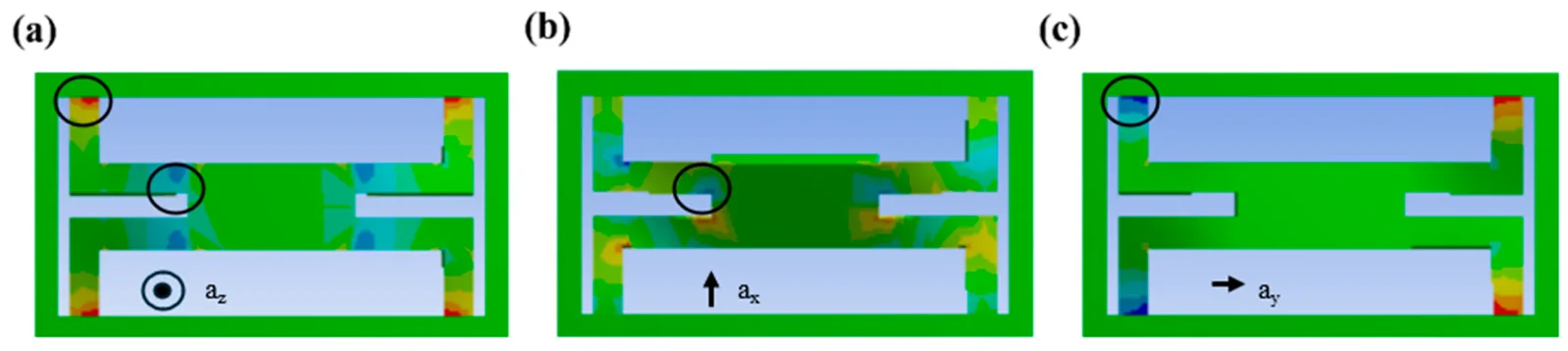
Strain distributions of the initial structure under a 1-g acceleration applied along the (**a**) *Z*-, (**b**) *X*-, and (**c**) *Y*-axis. The outer frame was fixed, and the coordinate system is indicated in each subfigure.

Although Ref. [10] achieved a structural triaxial design using a novel folded L-shaped configuration, it suffered from excessive cross-axis interference. Therefore, in this subsection, the structure from the literature is taken as the baseline and optimized step by step, aiming to reduce cross-axis interference while maintaining a comparable bandwidth and simultaneously enhancing sensitivity. The three-axis strain distribution of the initial structure is shown in Figure 3. As indicated by the circled areas, the strain distribution of the *Z*-axis overlaps with those of the *X*- and *Y*-axes, indicating a considerable level of cross-axis interference in this structure. The green color represents the zero-strain reference. As the color shifts toward either blue or red, the strain magnitude increases, with blue and red indicating strains in opposite directions.

**First-step optimization: increase the bandwidth**

**Figure 4:**
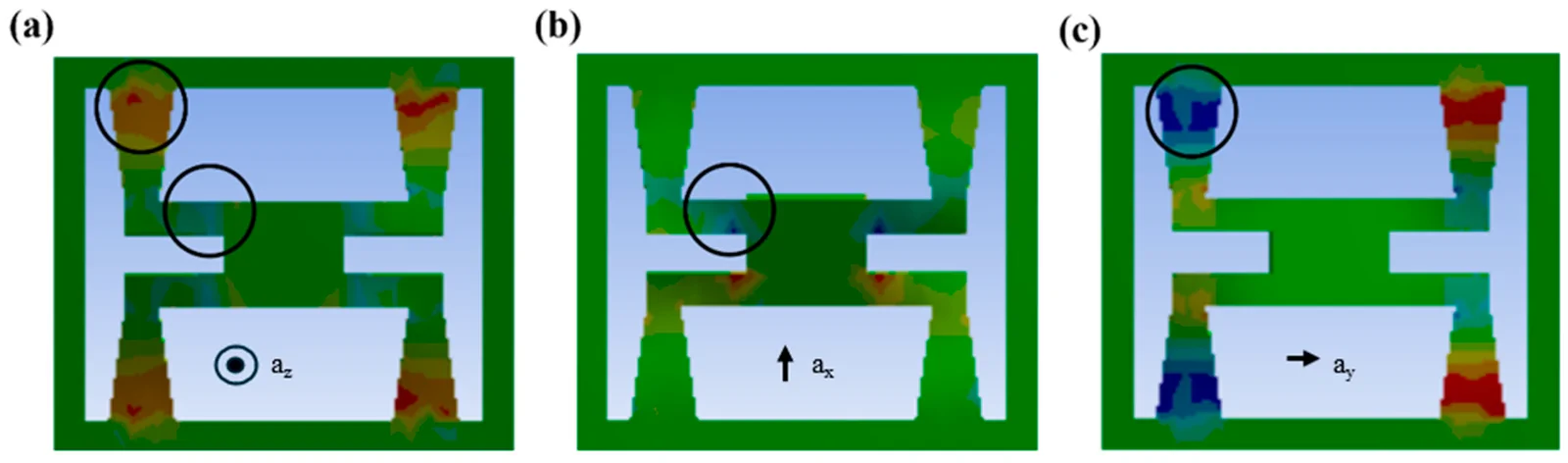
Strain distributions of the first-stage structure with trapezoidal suspension beams under a 1-g acceleration applied along the (**a**) *Z*-, (**b**) *X*-, and (**c**) *Y*-axis. The outer frame was fixed, and the coordinate system is indicated in each subfigure.

To prevent the subsequent optimization steps from significantly reducing the bandwidth, the original rectangular cantilever beams were modified into trapezoidal ones [14]. This optimization increases the beam width w in the resonance frequency formula derived in Section 2.2, where $f\propto \sqrt{w}$; therefore, this approach effectively enhances the accelerometer’s bandwidth. After optimization, the resonance frequencies of the *X*-, *Y*-, and *Z*-axes increased from 41 kHz, 23 kHz, and 12.7 kHz to 50.2 kHz, 31.2 kHz, and 17.3 kHz, respectively. However, as shown in Figure 4, the three-axis strain distribution of the trapezoidal cantilever beams still exhibits considerable cross-axis interference. The circled regions indicate the locations of the maximum strain amplitudes along the three axes. However, the regions of maximum strain for the *Y*- and *Z*-axes partially overlap.

**Second-step optimization: lower the cross-axis interference**

**Figure 5:**
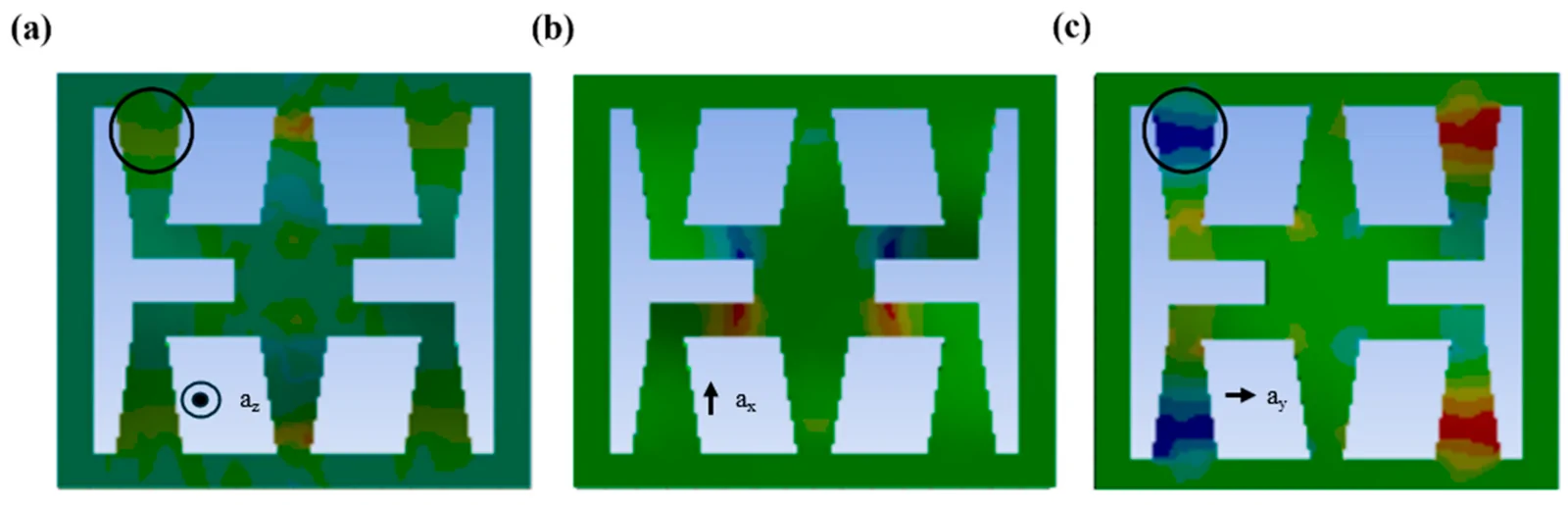
Strain distributions of the second-stage structure under a 1-g acceleration applied along the (**a**) *Z*-, (**b**) *X*-, and (**c**) *Y*-axis. The outer frame was fixed, and the coordinate system is indicated in each subfigure.

In the next step, two additional trapezoidal cantilever beams were added at the top and bottom of the accelerometer. The purpose of this modification was to transfer the strain that originally overlapped with the *X*- and *Y*-axes to these two beams, thereby achieving strain separation. Figure 5 shows the three-axis strain distribution of the accelerometer after adding the two trapezoidal cantilever beams. The overlapping region is now limited to a small area indicated by the circle, and the *Z*-axis strain in this region is light green, indicating that its magnitude is small. This result demonstrates that the addition of the two trapezoidal beams significantly reduces cross-axis interference and represents a crucial step toward realizing a truly structural triaxial accelerometer.

**Third-step optimization: increase the sensitivity**

**Figure 6:**
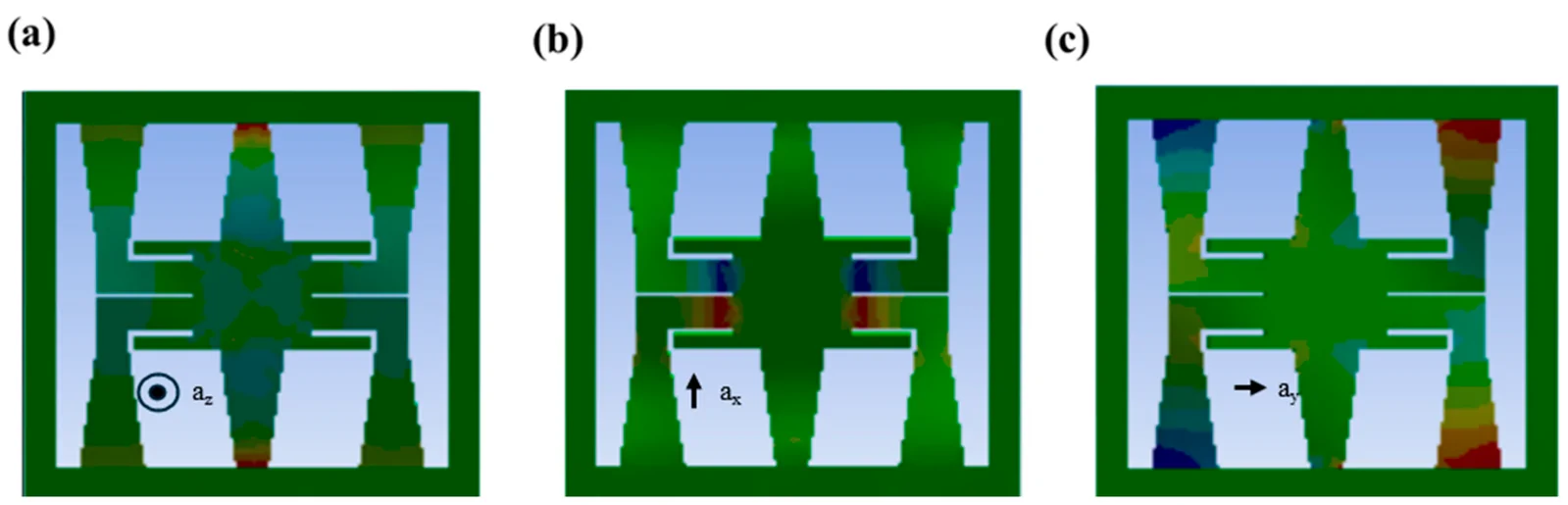
Strain distributions of the final structure with the modified four-bar proof mass under a 1-g acceleration applied along the (**a**) *Z*-, (**b**) *X*-, and (**c**) *Y*-axis. The outer frame was fixed, and the coordinate system is indicated in each subfigure.

**Figure 7:**
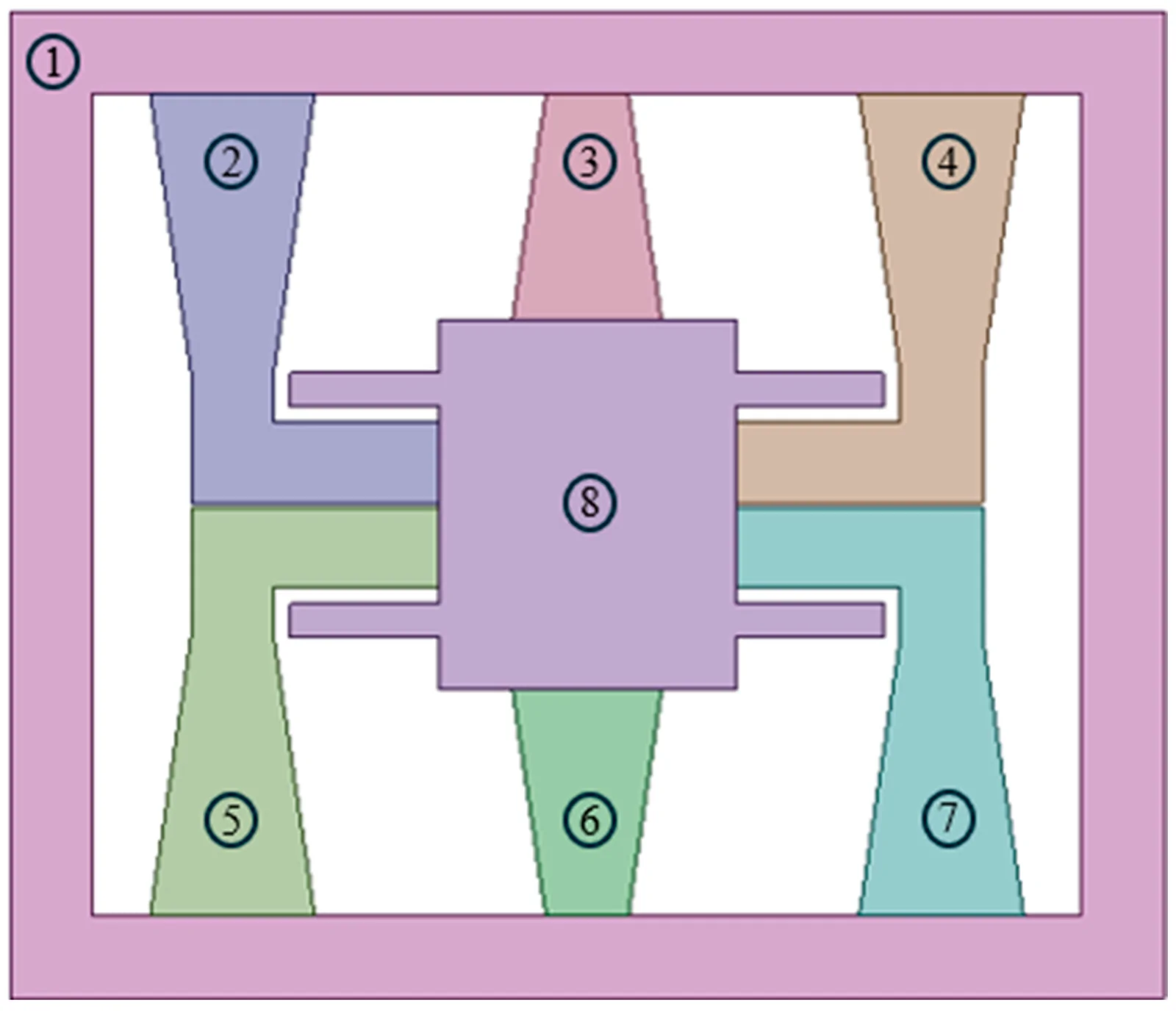
Top view of the final optimized accelerometer.

In the previous design, the strain areas for the *Z*- and *X*-axes were relatively small, and the regions of high strain intensity (shown in red and blue) were limited, suggesting lower sensitivity. Therefore, the proof mass was modified by adding four extended masses around its outer edges to enhance the *Y*-axis vibration while simultaneously improving the *Z*-axis response, thereby increasing the sensitivities of both the *Y*- and *Z*-axes. Figure 6 shows the three-axis strain distribution after the third optimization. For the three sensing axes, these high-strain regions are spatially separated and distributed at distinct locations, thereby effectively minimizing mutual interference among the *X*-, *Y*-, and *Z*-axis responses. Similar to the second optimization stage, the cross-axis interference is nearly eliminated, while the strain magnitudes along both the *Y*- and *Z*-axes are significantly enhanced. Figure 7 shows the top view of the final accelerometer design, which consists of the outer frame, the central proof mass, and the remaining cantilever beams. The numbered labels in the figure denote different structural components of the device. Region 1 represents the fixed frame, regions 2–7 correspond to the cantilever beam structures, and region 8 represents the proof mass. In future work, the device will be fabricated on a silicon wafer using MEMS processes.

### 3.3. Frequency Response Analysis

**Figure 8:**
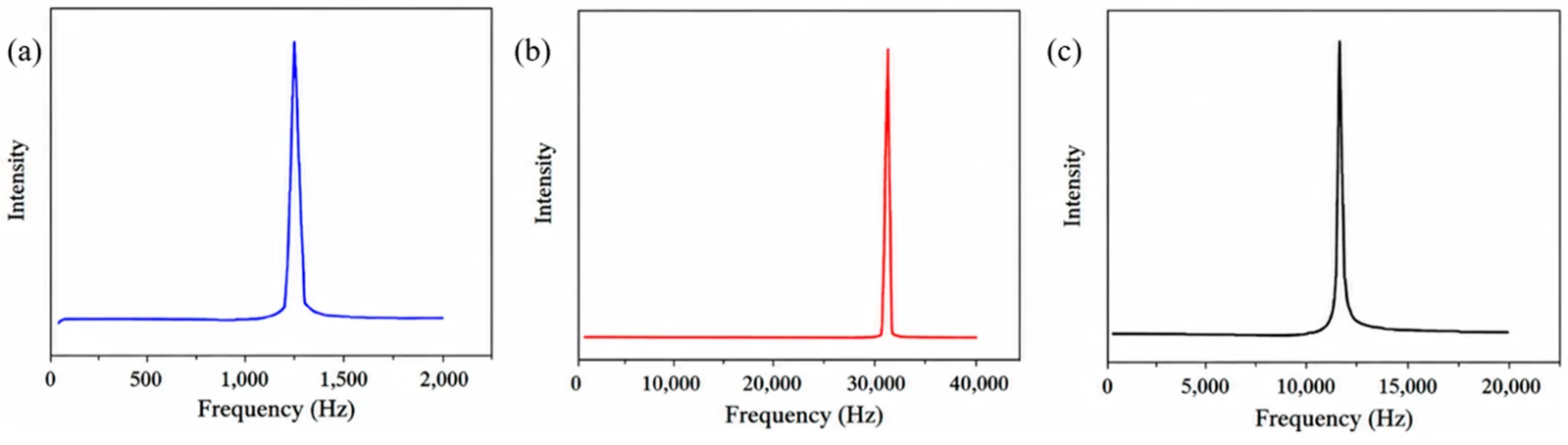
Frequency responses of the optimized accelerometer under harmonic base acceleration applied along the (**a**) *X*-, (**b**) *Y*-, and (**c**) *Z*-axis. The vertical axis represents the displacement amplitude obtained from the ANSYS simulation, with units of µm.

The three-axis frequency response of the optimized structure, simulated using ANSYS, is shown in Figure 8. The resonance frequencies of the *X*-, *Y*-, and *Z*-axes are approximately 12.6 kHz, 31.6 kHz, and 11.8 kHz, respectively, while those of the non-optimized structure are about 27.2 kHz, 38.4 kHz, and 11.3 kHz for the *X*-, *Y*-, and *Z*-axes, respectively. It can be observed that the resonance frequencies of the *X*- and *Y*-axes are lower than those of the non-optimized structure and gradually approach that of the *Z*-axis. According to the formulas derived in the previous section, a possible reason is that the modified proof-mass shape and the trapezoidal cantilever beams reduce the stiffness of each axis, leading to a decrease in the resonance frequencies of the *X*- and *Y*-axes. Although the overall three-axis resonance frequencies and bandwidth of the accelerometer decrease after optimization, the *Z*-axis resonance frequency increases. This indicates that the applicable range of the optimized structure does not differ significantly from that of the original design, showing no substantial change in bandwidth.

### 3.4. Sensitivity Analysis

**Figure 9:**
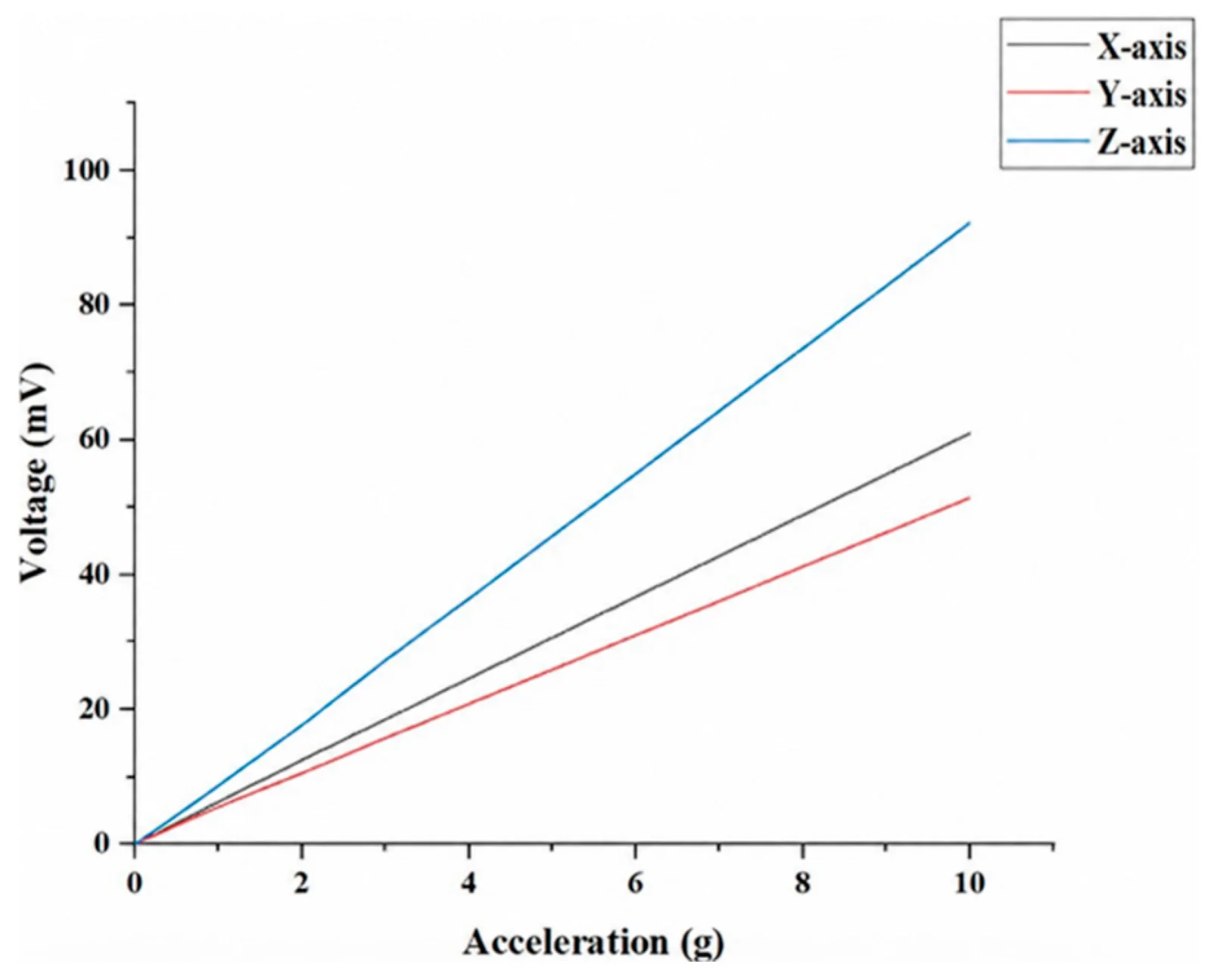
Voltage–acceleration response of the accelerometer.

The relationship between voltage and acceleration of the accelerometer is shown in Figure 9. The slope of each curve represents the sensitivity of the corresponding axis. The sensitivities of the *X*-, *Y*-, and *Z*-axes are approximately 6.11, 5.44, and 8.85 mV/g, respectively, whereas those of the non-optimized structure are about 0.66, 1.07, and 3.35 mV/g. After optimization, the sensitivities not only become more balanced among the three axes but also increase significantly.

### 3.5. Cross-Axis Analysis

**Figure 10:**
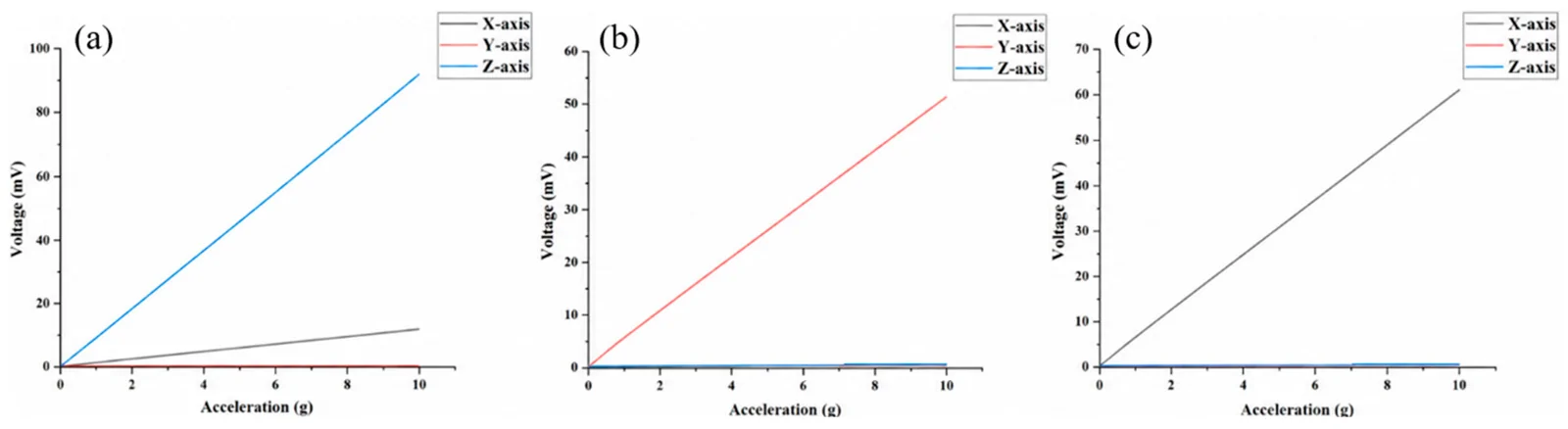
Ratio of cross-axes. (**a**) *Z*-axis excitation, (**b**) *X*-axis excitation, (**c**) *Y*-axis excitation

Although both bandwidth and sensitivity are improved after structural optimization, the most critical enhancement lies in the improvement of cross-axis interference. As described in the previous subsection, the drawback of the non-optimized structure is that the measured axis signals are significantly affected by excessive cross-axis interference, resulting in large measurement errors. The three-axis strain distribution of the initial structure is shown in Figure 3. It can be observed that, as indicated by the circled areas, the strain distribution of the *Z*-axis overlaps with those of the *X*- and *Y*-axes, indicating a considerable level of cross-axis interference in this structure. The three-axis strain distribution of the optimized structure is shown in Figure 6. It can be observed that the strain distributions of the three axes exhibit almost no mutual interference and cover a larger area, which is consistent with the significant improvement in sensitivity. Figure 10 shows a schematic representation of the cross-axis interference. The three plots illustrate the voltage responses of the three-axis piezoelectric accelerometer under acceleration applied along the *X*-, *Y*-, and *Z*-axes, respectively. In each case, the corresponding sensing axis exhibits the dominant and approximately linear voltage response, while the outputs from the other two axes remain relatively small, indicating good axis selectivity and reduced cross-axis interference. The blue line represents the measured signal, while the black line indicates the contribution ratio of other axes to the measured sensitivity. The measured signal value is normalized to 1, and the black line is presented as a relative value. Among the three axes, the *Z*-axis signal is the least accurate, as it contains approximately 15% contributions from the *X*- and *Y*-axis signals, whereas the other two axes each contain less than 10%. Therefore, structural optimization significantly reduces cross-axis interference and measurement error.

## 4. Conclusions

This study focuses on the mathematical modeling, simulation, and structural optimization of a MEMS three-axis piezoelectric accelerometer, a configuration that remains relatively uncommon among current commercial accelerometer devices. The main objective is to achieve high sensitivity and low cross-axis interference while maintaining a wide operational bandwidth. To this end, a mathematical model of the initial unoptimized L-shaped structure was first established and validated through comparisons with the reported literature, finite-element simulations, and analytical predictions. Based on this validated model, a systematic three-stage structural optimization was carried out.

The first optimization step replaces the original rectangular cantilever beams with trapezoidal beams. By increasing the effective beam width, the resonant frequency is raised, providing sufficient bandwidth margin for subsequent design modifications. In the second step, two additional trapezoidal suspension beams are introduced at the top and bottom of the structure. These beams guide and redistribute the strain that originally overlapped among the *X*-, *Y*-, and *Z*-axis sensing regions, thereby achieving physical strain separation. As a result, the off-diagonal coupling terms in the equivalent stiffness matrix are effectively suppressed, and cross-axis interference is significantly reduced. In the final step, the proof mass is redesigned into a 4-bar proof-mass structure by adding four extended masses around its outer edges. Rather than simply increasing the mass or softening the beams, this design redistributes the strain energy and enlarges the high-strain regions in the piezoelectric layer, resulting in a substantial enhancement of sensitivity. A comparison between the optimized design and previously published works is summarized in Table 3, demonstrating that the proposed accelerometer not only achieves higher resonant frequencies but also provides significantly greater sensitivity under comparable bandwidth conditions.

A key finding of this work is that sensitivity enhancement can be achieved without sacrificing bandwidth, which departs from the conventional trade-off between sensitivity and resonant frequency. After optimization, the resonant frequencies along the *X*-, *Y*-, and *Z*-axes are approximately 12.6 kHz, 31.6 kHz, and 11.8 kHz, respectively. Meanwhile, the corresponding sensitivities increase significantly from 0.66, 1.07, and 3.35 mV/g to 6.11, 5.44, and 8.85 mV/g. In particular, the *Z*-axis resonant frequency increases from 11.3 kHz to 11.8 kHz while its sensitivity is simultaneously improved, demonstrating that the proposed structural design enhances strain utilization rather than relying on the traditional stiffness–sensitivity trade-off.

Furthermore, the proposed design reduces cross-axis interference to below 15%, with the *X*- and *Y*-axis coupling further suppressed to below 10%. This improvement is achieved through structural strain separation rather than external circuit compensation. Therefore, the optimized accelerometer realizes high sensitivity, wide bandwidth, and low cross-axis coupling within a single integrated three-axis sensing structure.

Overall, this work evolves the conventional L-shaped accelerometer into a composite structure consisting of a W-shaped compound cantilever and a four-bar proof mass, achieving enhanced sensitivity and competitive resonant frequencies under comparable bandwidth conditions. These results demonstrate the potential of the proposed MEMS three-axis piezoelectric accelerometer for vibration and condition-monitoring applications. Future work will focus on device fabrication and experimental validation. A feasible process may include PZT thin-film and electrode deposition on a silicon substrate, lithographic patterning, DRIE-based formation of the cantilever and proof-mass structures, and subsequent wafer bonding or packaging. The fabricated device will be characterized in terms of resonance frequency, voltage sensitivity, and cross-axis response to assess the influence of fabrication-related non-idealities. Further studies will also investigate model refinement, multi-objective structural optimization, and experimental calibration to reduce residual cross-axis coupling and improve overall device performance.

## Figures and Tables

**Table 1:** Material parameters used in the simulation.

PZT [13]		Si [10]	
Density	7750 (kg/m^3^)	Density	2320 (kg/m^3^)
Poisson	0.31	Poisson	0.278
Young’s Modulus	63 (GPa)	Young’s Modulus	160 (GPa)
d_33_	136.504 (pm/V)		
Dielectric constant	462.94		

**Table 2:** Comparison of the *X*-, *Y*-, and *Z*-axis resonant frequencies obtained from the literature, finite element simulation, and mathematical model.

	Resonant Frequency (kHz)		
	X (Torsion)	Y (Torsion)	Z (Flexure)
Ref [10]	58.5	47.6	11.7
Simulation	39.4	24.2	11.3
Math model	41	23	12.7

**Table 3:** Comparison of the resonant frequencies and sensitivities of tri-axis accelerometers.

Type	Resonant Frequency (kHz)			Sensitivity (mV/g)			Ref
	X	Y	Z	X	Y	Z	
cross	--	--	4.11	0.714	0.714	1.72	[8]
This study	12.6	31.6	11.8	6.11	5.44	8.85	--
Optimized L-shape	--	--	--	0.29	0.245	2.412	[11]
L-shape (ref paper)	58.5	47.6	11.7	1.07	0.66	3.35	[10]
cross	0.381			7.398	7.398	53.976	[9]

## Data Availability

The data presented in this study are available on request from the corresponding author.
